# Case Study of a Young Adult With Ewing Sarcoma

**Published:** 2016-09-01

**Authors:** Carol Guarnieri

**Affiliations:** HonorHealth Scottsdale Shea Medical Center, Scottsdale, Arizona

## CASE STUDY

Maria was a 23-year-old woman who initially presented with a 1-year history of pain in the left heel. Over time, the pain had progressively worsened. Eventually, her foot had swollen, and she had difficulty walking, which required her to seek medical attention.

Initial imaging included a lower extremity magnetic resonance imaging (MRI) scan that revealed a malignant-appearing 5-cm lesion in the left calcaneus. A core needle biopsy showed bone that was infiltrated with poorly differentiated round malignant cells that were positive for CD99, which is consistent with a diagnosis of Ewing sarcoma. A staging positron-emission tomography–computed tomography (PET-CT) scan demonstrated a moderate hypermetabolic primary malignant lesion with a standardized uptake value (SUV) of 5.7 of the left calcaneus, as shown in the Figure below. The result also showed multiple mild hot spots in the bony skeleton, including the lumbar spine at L3 and L5 vertebral bodies, the right femur, and the left femur, suspicious for metastatic disease. In addition, the PET-CT showed numerous small lung nodules that were too small to characterize. Maria denied a fever or weight loss but did complain of fatigue. Her laboratory values were within normal limits.

**Figure:**
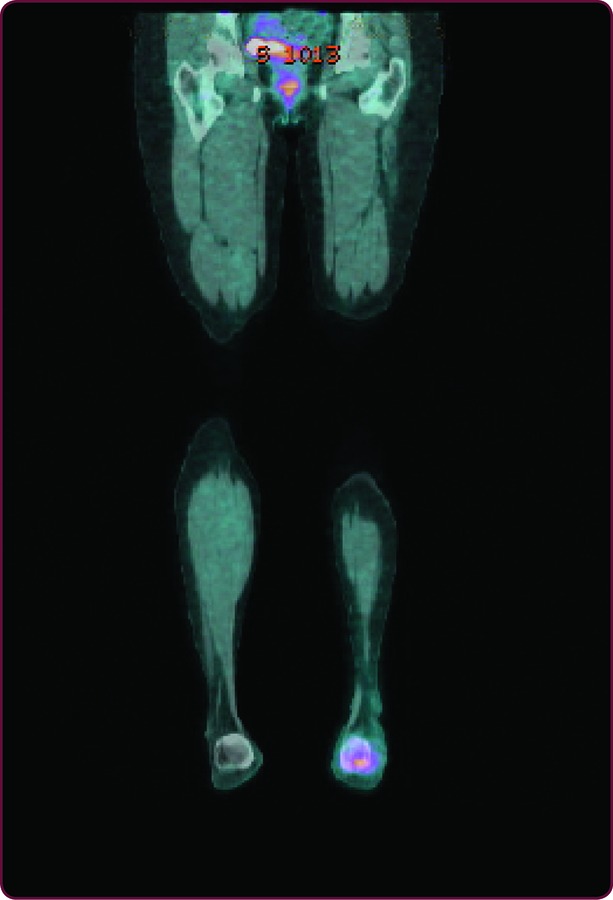
This staging PET-CT scan shows a moderate hypermetabolic primary malignant lesion in the left calcaneus.

At our hospital’s sarcoma tumor board meeting, a multidisciplinary team discussed the treatment plan for all patients with sarcoma. For Maria, we decided that based on her PET-CT scan, she had metastatic disease. We agreed it was best to treat her systemically with combination chemotherapy. In preparation of anthracycline chemotherapy, a two-dimensional echocardiogram was completed, and it showed an ejection fraction of 60%. We also discussed fertility preservation with Maria and her husband. They chose not to have egg preservation, but she started on monthly leuprolide injections. She was admitted to the inpatient oncology unit to begin chemotherapy, which included cyclophosphamide, doxorubicin, and vincristine with alternating cycles of ifosfamide and etoposide.

##  

Sarcoma comes from the Greek word Sarx meaning "flesh." Sarcomas are divided into main categories of bone sarcomas and soft-tissue sarcomas. In 1921, James Ewing described a primary bone tumor composed of small round blue cells that was histologically different from osteosarcoma. Today, the Ewing sarcoma (ES) family of bone tumors includes primitive neuroectodermal tumors (PNET), Askin’s tumor, and extraosseous ES (National Comprehensive Cancer Network [NCCN], 2015a).

Ewing sarcoma is the second most common type of primary bone cancer, accounting for approximately 25% to 34% of malignant bone tumors (DuBois, Grier, & Lessnick, 2009). The incidence of ES has remained unchanged for 30 years. The median age of patients with ES is 15 years, and more than 50% of patients are adolescents. Ewing sarcoma is uncommon in people of African American and Asian descent (Marina, 2010). The etiology of ES is unknown.

Ewing sarcoma can arise from any bone, but the primary sites include the long bones of the lower extremities and the bones of the pelvis, followed by the chest wall. Tumors of the hands and feet and head develop only rarely, accounting for up to 10% of all tumors (Wu & Montgomery, 2008). Patients with ES exhibit local symptoms such as pain, swelling, and tumor mass formation. Patients usually seek treatment due to these reasons. Unlike patients with other bone sarcomas, patients may also be seen with constitutional symptoms such as fever, weight loss, and fatigue. Other systemic signs may include anemia and an elevated level of lactate dehydrogenase (LDH), elevated erythrocyte sedimentation rate (ESR), and leukocytosis (Bacci et al., 2004).

## DIAGNOSTIC WORKUP

If ES is suspected, the workup should include magnetic resonance imaging (MRI) of the primary site. On MRI, bone lesions most often arise in the diaphysis or metaphyseal region. Classically, the periosteum demonstrates an "onion skin" appearance (Doroshow, 2016). CT (computed tomography) of the lungs should be done to evaluate for metastatic disease. A bone scan can also be performed to screen for other sites of metastatic disease. A PET (positron-emission tomography) scan is a valuable tool for staging and restaging of ES, with 96% sensitivity and 92% specificity (Treglia et al., 2012). A bone marrow biopsy should be considered to detect disease in the bone marrow. Laboratory tests include a complete blood cell count with a differential, complete metabolic panel, LDH, and ESR.

A controversial issue concerning tumor contamination in the biopsy path is of real concern. A review of the literature looking at neoplasm seeding in biopsies performed on the musculoskeletal system revealed that without resection of the biopsy tract, the possibility of local recurrence is real. Therefore, biopsies should be referred to a surgical oncologist trained in proper biopsy technique (Oliveira, Lima, da Silva, & de Mello, 2014).

Cytogenetic and/or molecular studies of the biopsy specimen should be done. The t(11;22)(q24;q12) translocation is found in 85% of Ewing cases, resulting in fusion of portions of the *EWSR1* gene on chromosome 22 with portions of the *FLI1* gene on chromosome 11 (Ghisoli et al., 2015). Other translocations have also been identified. The *EWS* gene can also fuse with a gene closely related to *FL1*. More research is needed to learn about the potential prognostic significance of the different translocations. Immunohistochemistry plays a key role in differentiating ES from other small round cell tumors. The most useful antigen is CD99. This protein is part of the *MIC2* gene and is found in more than 90% of cases of ES (DuBois et al., 2009).

## PROGNOSTIC INDICATOR

The presence of metastatic disease at initial diagnosis is widely regarded as the most important prognostic indicator. Patients with metastatic disease at presentation had a 5-year survival of 21% compared with 55% for nonmetastatic patients (Cotterill et al., 2000). Approximately 20% to 30% of patients will present with metastatic disease at the time of diagnosis. The most common site of metastatic disease is the lungs, followed by bone and bone marrow. Metastatic disease to the lungs carries a better prognosis than bone-only disease (Blaney et al., 2001). In addition, response to the tumor with neoadjuvant chemotherapy has also been shown to play a role in predicting outcome. Patients who demonstrated complete tumor necrosis have a better disease-free survival (Anderson, Randal, Springfield, & Gebhardt, 2014).

## CURRENT TREATMENT

**Local Disease**

In the 1960s, surgery and radiation therapy were the main treatments of ES. Prior to the use of chemotherapy, almost all patients with ES died of distant metastatic disease (Anderson, Randal, Springfield, & Gebhardt, 2014). Currently, the treatment of ES involves a multidisciplinary evaluation and treatment protocols using chemotherapy, surgery, and radiotherapy. Preservation of function and reduction of long-term complications should be taken into consideration in determining local tumor control for patients.

Improved combinations of neoadjuvant chemotherapy result in a marked reduction in tumor volume, often improving the feasibility of surgical resection and reconstruction. Multiagent chemotherapy regimens including ifosfamide, cyclophosphamide, doxorubicin, etoposide, dactinomycin (actinomycin), and vincristine have been shown to be effective in patients with ES (NCCN, 2015a). With the use of neoadjuvant chemotherapy, the outcome of limb-salvage surgery has improved, allowing surgeons to achieve functional reconstructions. Adjuvant chemotherapy following wide excision or amputation is recommended for all patients regardless of surgical margins. The duration of chemotherapy should be between 28 to 49 weeks, depending on the dose schedule (Burgert et al., 1990).

Ewing sarcoma is extremely radiosensitive. Definitive radiation therapy given with concurrent chemotherapy can be an effective treatment option for patients who have unresectable tumors. Radiation therapy can also be given postoperatively for tumors resected with positive margins (Ozaki, 2015). Furthermore, patients with metastatic disease are more likely to receive radiation therapy to provide local control of their disease.

**Metastatic/Relapsed Disease**

Patients who present with metastatic disease have a poor outcome. These patients appear to have more chemotherapy-resistant disease. The goal of therapy is to control disease with individualized treatment. Various strategies are considered for these patients, ranging from palliative care to more aggressive multimodality therapy. Other chemotherapy regimens that include topotecan, irinotecan, carboplatin, and temozolomide have been used, with response rates ranging from 10% to 30% (Casey et al., 2009).

The prognosis for patients with recurrent disease is poor. Recurrence involving distant sites is more common than isolated local recurrence. The time from diagnosis to relapse appears to be the strongest determinant of outcome following relapse. For patients with an early recurrence (less than 2 years from initial diagnosis), the likelihood of long-term disease control is less than 10% (Barker, Pendergrass, Sanders, & Hawkins, 2005). In one large group cooperative analysis, median overall survival from the time of recurrence was 14 months (Shankar, Ashley, Craft, & Pinkerton, 2003).

Retrospective studies have evaluated the role of myeloablative therapy and autologous stem cell rescue for metastatic/recurrent disease, with equivocal results (Ladenstein et al., 2010). These patients should be offered participation in clinical trials evaluating novel approaches to therapy.

## FUTURE TREATMENT

Since 85% of patients with ES have the t(11;22)(q24;q12) translocation, this fusion has been a target for new therapies. It has become clear that this fusion is necessary for tumor growth, and targeting the mutant transcription itself or downstream targets is an attractive therapeutic strategy. However, direct inhibition of the fusion has proved to be difficult. Drugs targeting the *EWS-FLI1* gene in clinical trials include several multikinase inhibitors, poly(ADP-ribose) polymerase (PARP) inhibitors, and mithramycin (Arnaldez & Helman, 2014).

Although historically sarcomas as a whole have shown disappointing clinical immunoresponsiveness, recent research and clinical findings have led to renewed enthusiasm for immunotherapy. Preclinical studies have shown the effectiveness of cytotoxic CD8 T-cell targeting the *EWS-FLI1* fusion gene–specific expressed antigens.

In a phase I study in 12 patients with ES, FANG immunotherapy, using an autologous tumor cell vaccine given monthly for 4 months by injection, showed promising results. The therapy was well tolerated, elicited a tumor-specific immune response in all patients, and was associated with a favorable 1-year survival (Ghisoli, et al., 2015). Further clinical testing is indicated.

In addition, to better define the genetic landscape of ES, researchers, using modern sequencing techniques, found ES has a genome that is less complex than other cancers. Ewing sarcoma was frequently affected by mutations in*STAG2*, a gene that has recently gained attention due to its importance in the biology of several cancer types. It was also shown that patients with ES whose tumors were affected by *STAG2* loss may have a worse prognosis (Brohl et al., 2014). Helping to define the genetic landscape of ES will also provide a starting point for individualized care in this cancer.

## CARING FOR ADOLESCENTS/YOUNG ADULTS

The duration of treatment for patients with ES is usually complex and long. Caring for adolescents and young adults (AYAs) with cancer can be a unique challenge for advanced practitioners (APs), who are used to caring primarily for an adult population. Adolescents and young adults diagnosed with cancer should be recognized as a distinct age group with unique medical and psychosocial needs (NCCN, 2015b). A diagnosis of cancer can affect critical developmental activities of AYAs. Missing school or work; losing normal life experiences such as dating; needing to be protective of one’s health; being seen or seeing oneself as different; and/or dealing with issues of grief, loss, and death if cancer becomes untreatable create isolation and alienation from peer groups (Samuel, 2011). If APs do not understand the unique needs of AYAs, they risk alienating patients through ignorance—and alienation is the enemy of trust and communication.

Although there are many descriptive studies of AYAs with cancer, there are few intervention studies on how to help AYAs with cancer to cope (Katz, 2015). However, a systematic review and meta-analysis of 452 manuscripts among pediatric, adolescent, and young adult bone tumor survivors found that quality-of-life (QOL) studies are remarkably diverse, making it difficult to detect trends in patient outcomes. Prospective studies suggest QOL improves over time, and females and patients who are at an older age at the time of diagnosis tend to have poor QOL (Stokke, Sung, Gupta, Lindberg, & Rosenberg, 2015).

Once treatment is complete, a critical aspect of care for APs is working together with AYAs to develop a personalized follow-up care plan. Participating in survivorship care helps many survivors feel in control as they transition back to their lives prior to their cancer diagnosis. Keeping a medical support system in place is essential for maintaining physical and psychosocial health.

Supportive care interventions aimed at AYAs can assist APs in optimizing treatment to impact patient outcomes. In collaboration with other health-care professionals, APs can lead the team in ensuring the needs of AYAs are met. Supportive care tools are provided in the Table.

**Table:**
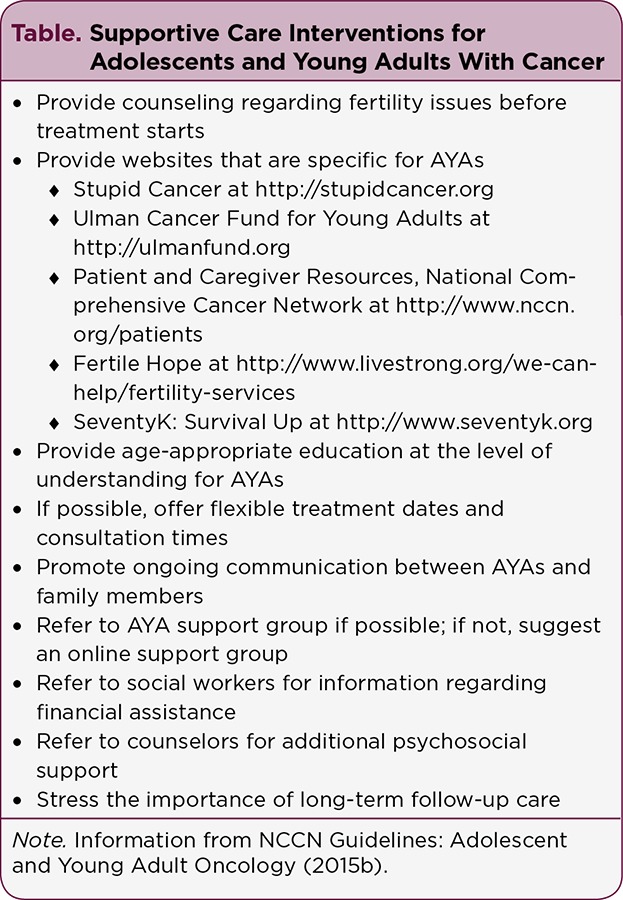
Supportive Care Interventions for Adolescents and Young Adults With Cancer

## CONCLUSION

Maria completed her initial chemotherapy without complications. Three months after her chemotherapy was completed, she complained of increased pain in her lumbar spine. Restaging imaging showed progression of disease. She received palliative radiation therapy and additional chemotherapy with minimal response to treatment. Maria wanted to continue to fight her cancer and her family was very supportive of her decision. She was referred to a major cancer center where she entered a clinical trial and unfortunately died of her disease shortly thereafter.

Advanced practitioners are essential in assisting AYAs to cope with this challenging diagnosis, and multidisciplinary teams with APs play a crucial role in further improving patient outcomes. The prognosis of patients with ES has improved in recent years. With the advancement of neoadjuvant chemotherapy, limb-sparing surgeries have become more frequent. This treatment has improved not only prognosis but QOL for patients with a diagnosis of ES. Further research into cytogenetic abnormalities may show the promise of novel targeted therapies for ES.
